# Biological Control of Tephritid Fruit Flies in Argentina: Historical Review, Current Status, and Future Trends for Developing a Parasitoid Mass-Release Program

**DOI:** 10.3390/insects3030870

**Published:** 2012-09-14

**Authors:** Sergio M. Ovruski, Pablo Schliserman

**Affiliations:** Biological Control Division, Pilot Plant of Industrial Microbiological Processes and Biotechnology, Avda. Belgrano y Pje. Caseros, T4001MVB, S. M. de Tucumán, Argentina; E-Mail: schliserman73@yahoo.com.ar

**Keywords:** Argentina, parasitoids, fruit flies, biological control, *Anastrepha fraterculus*, *Ceratitis capitata*

## Abstract

In Argentina there are two tephritid fruit fly species of major economic and quarantine importance: the exotic *Ceratitis capitata* that originated from Southeast Africa and the native *Anastrepha fraterculus*. In recent years, the use of fruit fly parasitoids as biocontrol agents has received renewed attention. This increasing interest has recently led to the establishment of a program for the mass rearing of five million *Diachasmimorpha longicaudata *parasitoids per week in the BioPlanta San Juan facility, San Juan, Argentina. The first augmentative releases of *D. longicaudata* in Argentina are currently occurring on commercial fig crops in rural areas of San Juan as part of an integrated fruit fly management program on an area-wide basis. In this context, research is ongoing to assess the suitability of indigenous parasitoid species for successful mass rearing on larvae of either *C. capitata *or *A. fraterculus*. The purpose of this article is to provide a historical overview of the biological control of the fruit fly in Argentina, report on the strategies currently used in Argentina, present information on native parasitoids as potential biocontrol agents, and discuss the establishment of a long-term fruit fly biological control program, including augmentative and conservation modalities, in Argentina’s various fruit growing regions.

## 1. Introduction

In Argentina, tephritid fruit flies of economic importance are only represented by the introduced Mediterranean fruit fly (medfly), *Ceratitis capitata *(Wiedemann), and the native South American fruit fly, *Anastrepha fraterculus *(Wiedemann). Fruit infestation levels by these two tephritid species varies between 15% and 20% of overall Argentinean fruit production, which represents a reduction of profit margins nationwide of up to approximately US$ 90 million per annum [1,2]. In addition, the presence of both *C. capitata* and *A. fraterculus* constrains fruit crop diversification plans made by local governments and severely limits fresh fruit exports due to quarantine restrictions imposed by importing countries free of these two pest species or with active control programs [2]. Among the tephritid fruit flies, *C. capitata* is the most polyphagous species known [3] and one of the most important cultivated fruits pests in the world [4]. In Argentina, *C. capitata* infestations have been reported in at least 22 exotic fruit species of commercial value and in all of the country’s fruit growing regions [5,6]. *Anastrepha fraterculus* is one of the most polyphagous species of the *Anastrepha *genus, but its status as a pest varies geographically within the American continent [7]. For example, some of the cryptic South American species of the *A. fraterculus* group [8] cause serious economic damage to several *Citrus *spp. in Argentina, Brazil, and Colombia, whereas *A. fraterculus* is not considered to be a pest in the citrus-growing areas of Mexico [7]. Although *A. fraterculus* attacks commercial fruit species, it has mainly been reported infesting exotic and native fruit species in Argentina [5,9].

Historically, control of *C. capitata* and *A. fraterculus* in Argentina was attempted almost exclusively through the use of a mixture of insecticides (generally malathion) and food attractants (such as hydrolyzed corn protein, sugarcane molasses, or hydrolyzed soybean protein) commonly known as bait sprays [1,10]. Cultural practices, such as the removal of heavily infested fruit, have also been used [11]. There is currently an increasing interest in Argentina in combating both the medfly and the South American fruit fly through campaigns in which conventional chemical methods are gradually being substituted by ecological practices, such as the sterile insect technique (SIT), the use of specific lures and baits for trapping pestiferous flies, and new biological control strategies within integrated regional fruit fly management programs. These trends are mainly motivated by issues concerning human health and environmental safety including the globalization of markets and bans on many of the most effective insecticides. Fortunately, the use of fruit fly parasitoids as biocontrol agents is currently receiving renewed attention in Argentina. This increasing interest is mainly related to: (1) the improvement of mass rearing techniques for exotic and native parasitoids that allow the development of new control strategies involving augmentative releases of these natural enemies in several countries [12,13,14,15,16,17]; (2) the recent discoveries in North Africa of new, more specific parasitoids of *C. capitata*, including *Fopius ceratitivorus* Wharton and *F*. *caudatus *(Szépligeti) [18,19,20]; and (3) the present drive towards the conservation of biodiversity in agroecosystems through ecologically acceptable tactics, including the use of natural enemies [21,22,23].

Therefore, the objectives of this contribution are: (1) to comment on the fruit fly control methods and strategies currently being developed or utilized in Argentina; (2) to provide a detailed historical review of the fruit fly biological control programs developed during the past 80 years in Argentina; (3) to describe the current status of the biological control of fruit flies; and (4) to discuss the establishment of a long-term, well-planned fruit fly biological control program, including augmentative and conservation modalities, in Argentina’s various fruit growing regions.

## 2. Current Fruit Fly Control Strategies

The current expansion of the “National Fruit Fly Control and Eradication Programme” (ProCEM) of the National Agri-Food and Animal Health and Quality Service (SENASA) has led to the integration of actions against both *C. capitata* and *A. fraterculus* on a regional level in Argentina. These actions have allowed the division of the country into several fruit-producing regions based on their geographical and ecological conditions as well as the soils, distances and isolation characteristics of these areas. This has permitted the application of specific strategies for the control and eradication of one or both tephritid species of economic importance [2,24]. 

Currently, *C. capitata* is found throughout all Argentinian fruit-growing regions, covering latitudes from 22° to 56°S. In the cold and dry southern (Patagonia) and central-western (Cuyo) regions, where pome fruits (apple, pear, grape, quince) and stone fruits (peach, plum, apricot) are mainly grown, the only economically important tephritid species is *C. capitata*. In contrast, the native species *A. fraterculus *is mainly restricted to the warm and humid northwestern (NW) and northeastern (NE) regions (from 22° to 34°S latitude), which are largely citrus growing areas. In both northern regions, *A*. *fraterculus* and *C*. *capitata* naturally coexist on wild and commercially grown fruits.

In the Patagonia region and some fruit-producing highland valleys of the Mendoza and San Juan Provinces (Cuyo region), area-wide control actions against *C. capitata* based on the integrated use of SIT, selective types of toxic bait, cultural control methods, and quarantine systems, have resulted in either fruit fly pest-free areas or areas of low pest prevalence [2]. Several, large fruit growing areas in the Cuyo region remain a focus for area-wide management, with the aim to establish *C. capitata*-free and/or low prevalence areas and to ensure their continuation. In the citrus-producing areas of the NW and NE regions, both pestiferous fruit fly species are currently controlled by growers who exclusively use environmentally unfriendly methods, such as insecticides applied by air or ground sprays. In spite of the use of chemical control, the high levels of fruit damage recorded in citrus-growing areas each year demonstrate the urgent need to address this problem with an integrated approach to fruit fly control throughout the region. For this reason, the current goal of the expanded ProCEM plan is to improve fruit fly control in areas where *C. capitata*/*A. fraterculus* have a direct impact on *Citrus* spp. production by shifting from the use of chemical control at an orchard level to the integration of different technologies and strategies based on the area-wide management concept [2,24].

## 3. Fruit Fly Biological Control

### 3.1. Historical Overview

The available information on the natural enemies used in the biological control of tephritid pests in Argentina pertains to specific hymenopterous parasitoids, including both native and introduced exotic species.

#### 3.1.1. Native Parasitoids

The subtropical rain forests of NW and NE Argentina, locally known as “Yungas” and “Paranaense y de Galería” forests, respectively, support native parasitoid populations mainly associated with fruit flies of the *Anastrepha* genus. As a result of several surveys of fruit fly parasitoids performed over the past 75 years in these rain forests, 13 neotropical parasitoid species have been recorded in Argentina, most of which are associated only with *A. fraterculus *on native and exotic host plant species [25,26,27,28]. The most important parasitoid guild found in Argentina comprises a large group of koinobiont endoparasitic braconids and figitids that oviposit in host larvae and emerge from the puparium (Table 1). Two idiobiont pupal parasitoid guilds, whose species attack the fly host after pupation in the soil, have also been identified in Argentina. One of the two guilds is composed of only two indigenous endoparasitic diapriids whereas the other guild is comprised of the cosmopolitan species *Pachycrepoideus vindemmiae* (Rondani) and two other unidentified ectoparasitic pteromalid species (Table 1). No egg parasitoids (ovipositing in and emerging from the host egg) or egg-prepupal parasitoids (ovipositing in the host egg and emerging from the puparium) associated with tephritid fruit flies have been found in Argentina.

**Table 1:** Key biological characteristics of neotropical and cosmopolitan parasitoid species associated with *Anastrepha fraterculus* and *Ceratitis capitata* in Argentina. [25,26,27,28].

Parasitoid species and families	Parasitism modes ^4^	Feeder types ^5^	Host stage attacked ^6^	Host fruit fly species
BRACONIDAE				
*Asobara anastrephae *(Muessebeck) ^1^	S, K	En	L3	Af
*Doryctobracon areolatus *(Szépligeti) ^1^	S, K	En	L2–L3	Af, Cc ^8^
*Doryctobracon brasiliensis *(Szépligeti) ^1^	S, K	En	L3	Af, Cc ^8^
*Doryctobracon crawfordi *(Viereck) ^1^	S, K	En	L3	Af
*Opius bellus* Gahan ^1^	S K	En	L3	Af
*Utetes anastrephae* (Viereck) ^1^	S, K	En	L3	Af, Cc ^8^
FIGITIDAE				
*Aganaspis pelleranoi* (Brèthes) ^1^	S, K	En	L3	Af, Cc
*Dicerataspis grenadensis* Ashmead ^1^	S, K	En	L3	Af ^8^
*Lopheucoila anastrephae* (Rohwer) ^1^	S, K	En	L3	Af
*Rhoptromeris haywardi* (Blanchard) ^1^	S, K	En	L(?) ^7^	Af ^8^, Cc ^8^
*Odontosema anastrephae* Borgmeier ^1^	S, K	En	L3	Af
DIAPRIIDAE				
*Coptera haywardi* Loiácono ^1^	S, I	En	P	Af, Cc
*Tricopria anastrephae* Costa Lima ^1^	S, I	En	P	Af, Cc ^8^
PTEROMALIDAE				
*Pachycrepoideus vindemmiae* (Rondani) ^2^	S, I	Ec	P	Af, Cc
*Pachyneuron* sp. ^3^	S, I	Ec	P	Cc
*Spalangia* sp. ^3^	S, I	Ec	P	Cc

^1^ Neotropical origin. ^2^ Cosmopolitan. ^3^ Unknown origin. ^4^ S = solitary; K = koinobiont; I = idiobiont. ^5^ En = endoparasitoid; Ec = ectoparasitoid. ^6^ L2 = second instar larva; L3 = third instar larva; P = pupa. ^7^ Unknown larval stage. ^8^ Host fly-parasitoid association should be verified. Af, *A. fraterculus*; Cc, *C. capitata*.

The Argentinean rain forests represent an important source of parasitoid species that can potentially be used for the reduction of pestiferous fruit fly species. Isolated efforts have been made throughout the last century to implement biological control programs by increasing indigenous fruit fly parasitoids in the citrus-producing areas of northern Argentina. The first step towards obtaining and releasing native parasitoids was taken during the early 1930s using a simple method to catch parasitoids and flies in the NE province of Misiones [29]. This collection method consisted of digging a pit in the soil beneath the host plant into which fallen infested fruit was placed; the pit was then covered with a thin sieve only permitting the emergence of parasitoids, while the flies died inside the trap [29]. Additionally, during the late 1930s, native parasitoids were collected in wild vegetation and immediately released in several fruit orchards of the NW province of Tucumán using wooden cages with a detachable glass window [30,31]. Later, between the 1940s and 1980s, a method similar to Ogloblin’s parasitoid fly-trap model was used in commercial citrus crops in Tucumán [32]. However, more significant results have been achieved during the last 10 years (2002/2012) since the colonization and domestication of several neotropical parasitoid species occurring in Argentina [33,34,35]. Thus, the development of well-established native parasitoid colonies has enabled the implementation of extensive biological and ecological studies with the main objective of selecting candidate species for mass-rearing and augmentative release programs [33,34,36].

#### 3.1.2. Exotic Parasitoids

The introductions of exotic parasitoids into Argentina for fruit fly biocontrol are summarized chronologically in Table 2. In 1947 Argentina’ s Ministry of Agriculture and Cattle (MAG), together with Concordia’s Agricultural Research Station, introduced the eulophid parasitoid *Tetrastichus giffardianus* Silvestri to the country from Brazil for the control of *C. capitata*. This exotic species is a gregarious, larval-prepupal parasitoid that was initially introduced into Hawaii in 1913 from West Africa to control *C. capitata* [15] and was successfully established on all of the major Hawaiian Islands [17]. Later, *T. giffardianus* was shipped to various Latin America countries, including Brazil [37]. In Argentina, it was released in small numbers throughout citrus orchards in Tucumán and in the NE provinces of Corrientes and Entre Rios [38]. *Tetrastichus giffardianus* was recovered immediately after its release in NE Argentina [39], but there has been no evidence of permanent establishment at any release site. Although the introduction of *T. giffardianus* from Brazil represented an indirect release (*i.e.*, one country obtaining a particular species from a country into which it had previously been imported [40]), it can be categorized as the first classical fruit fly biological control program in Argentina.

**Table 2:** Chronological summary of the exotic parasitoid species introduced into Argentina for biological control of *Anastrepha fraterculus* and *Ceratitis capitata*. [11,27,37,38,39,40,41,42,43,44,45].

Introduction year(s)	Parasitoid species	Source ^1^ (institution and country)	Target host(s)	Successfully laboratory Reared	Parasitoid status			Releaseyear(s)	Totalnumber released
					Released	Recovered	Established		
1947	*Tetrastichus giffardianus *	IBSP, Brasil	*C. capitata*	No	Yes	Yes	No	1947	?
1961	Aceratoneuromyia *indica *	DGSV, Mexico	*A. fraterculus* **	Yes	Yes	Yes	Yes	1966–1969	700,000
			*C. capitata*					1973–1977	100,000
	*Diachasmimorpha longicaudata*	DGSV, Mexico	*A. fraterculus*	Yes	Yes	Yes	Yes	1966–1969	10,000
			*C. capitata*					1973–977	29,700
	*Pachycrepoideus* *vindemmiae* ^2^	DGSV, Mexico	*A. fraterculus*	Yes	Yes	Yes	Yes	1966–1969	96,000
			*C. capitata*					1973–1977	259,200
1966	*Fopius arisanus*	DGSV, Mexico	C. capitata	No	Yes	No	No	1966–1967	?
	*Doryctobracon crawfordi*	DGSV, Mexico	*A. fraterculus*	No	Yes	No	No	1966–1967	?
1977	*A. indica*	DGSV, Mexico	*A. fraterculus*	Yes	No	---	---	---	---
			*C. capitata*						
1986	*A. indica*	OIRSA, Costa Rica	*A. fraterculus* **	Yes	Yes	No	No	1986–1988	---
			*C. capitata*						
	*D. longicaudata*	OIRSA, Costa Rica	*A. fraterculus* **	Yes	Yes	No	No	1986–1988	8,000
			*C. capitata*						
	*P. vindemmiae ^2^*	OIRSA, Costa Rica	*A. fraterculus* **	Yes	Yes	Yes	---	1986–1988	3,000
			*C. capitata*						
1999	*Diachasmimorpha tryoni*	DGSV-Mexico	*C. capitata*	Yes	No	---	---	---	---
	*D. longicaudata*	DGSV-Mexico	*A. fraterculus* **	Yes	Yes	Yes	---	2012 to present	ca. 520,000
			*C. capitata*						

^1^ Institution names: DGSV = Plant Health Service—Moscamed and Moscafrut National Programs, Mexico; OIRSA = International Regional Organization for Plant and Animal Health; IBSP = Biological Institute of Sao Paulo, Brazil. ^2^ This table includes *P. vindemmiae*, although this cosmopolitan species was already present in Argentina before 1961 (see text).

In the 1960s, a new effort was implemented by MAG, together with the National Institute of Agriculture Technology (INTA), to control *C. capitata* and *A. fraterculus* by introducing five parasitoid species from laboratory colonies in Mexico [39]. The introduced species were the egg-prepupal parasitoid *Fopius arisanus *Sonan (reported as *Opius oophilus *Fullaway), the larval-prepupal parasitoids *Doryctobracon crawfordi* (Viereck) (reported as *Opius crawfordi *Viereck), *Diachasmimorpha longicaudata* (Ashmead) (reported as *Opius* or *Biosteres longicaudatus *Ashmead), and *Aceratoneuromyia indica* (Silvestri) (reported as *Syntomosphyrum indicum*) (all koinobiont endoparasitoids), and the pupal, idiobiont ectoparasitoid *P. vindemmiae*. All of these parasitoid species, with the exception of the neotropical parasitoid *D. crawfordi*, were previously shipped to Mexico from Hawaii [41]. In Argentina, laboratory colonies were established for three of these species (*D. longicaudata*, *A. indica* and *P. vindemmiae*) using *C. capitata* as a host. Specimens of these three parasitoid species were sporadically released in five Argentinian provinces [Tucumán and Jujuy (NW), Misiones and Entre Rios (NE), and Córdoba (Central region)] throughout the 1960s [38,39]. Both *D. longicaudata* and *A. indica* were recovered from *C. capitata* and *A. fraterculus* pupae immediately after releases at the following sites: Montecarlo (Misiones), San Miguel de Tucumán, Quebrada de Lules, El Siambón (Tucumán), Calilegua (Jujuy), Cruz del Eje and Yacanto (Córdoba) [42,43]. Parasitism of *A. fraterculus*/*C. capitata* larvae by *D. longicaudata* and *A. indica* was observed on guava and peach fruits, varying from 18.0% to 35.8% [38].

Recent surveys on fruit fly parasitoids performed in Misiones (NE) [44] and in Salta (NW) [45], included specimens of *D. longicaudata*. The first survey and record of this braconid since its release in the 1960’s was in March 2000, attacking *A. fraterculus* larvae associated with *Feijoa sellowiana* L. (Myrtaceae). This species was subsequently found in February and March 2001 and April 2002. Additional *D. longicaudata* specimens were obtained from *A. fraterculus* larvae infesting wild peaches between November and December 2001 in the locality of El Oculto, Salta. Similarly, *A. indica* was repeatedly recovered from *C. capitata* pupae between 1998 and 2001 in Córdoba and between 1999 and 2000 from *A. fraterculus* pupae in Misiones and Jujuy [43]. Thus, both *D. longicaudata* and *A. indica* were recovered approximately 40 years after their first releases in northern Argentina. Although *D. longicaudata* and *A. indica* were recovered in low numbers (≈1% parasitism), they can be considered to have been successfully established in Argentina as a direct result of early biological control programs.

Colonies of *F. arisanus* and *D. crawfordi* were not established in the laboratory in the 1960s, but these species were directly released in small numbers between 1966 and 1967 in citrus-growing areas of NW and NE Argentina. The egg-pupal parasitoid *F. arisanus* was released in Jujuy and Tucumán, but it was not recovered in the field, while *D. crawfordi* was released in Jujuy and in Misiones. However, permanent establishment of *D. crawfordi* was recently confirmed in the northwestern province of Salta [26]. Regarding the discovery of *D. crawfordi* in the locality of Orán in Salta, two probable interpretations are equally plausible: (1) Because *D. crawfordi* was introduced into Argentina and released in Calilegua and Ledesma, Jujuy (approximately 50 years ago), this braconid species may have been successfully established on *A. fraterculus* in the release area, and it could have spread to other neighboring areas of Northwest Argentina, such as Orán; (2) alternatively, considering that *D. crawfordi* is a widespread neotropical braconid species [37] and that it has been recovered in southern Bolivia, it is equally likely that the natural distribution range of this species includes NW Argentina.

A new biological control program was attempted in the 1980s by the Research Center for the Regulation of Noxious Organisms (CIRPON), Tucumán, Argentina, through the reintroduction of the widely distributed parasitoid species *D. longicaudata*, *A. indica* and *P. vindemmiae*. Initial shipments for this biocontrol program originated in Hawaii via Costa Rica. The program involved small laboratory colonies of these three parasitoid species and indirect releases of limited numbers of both *P. vindemmiae* and *D. longicaudata* in the NW provinces of Catamarca and Tucumán [39]. Unfortunately, the success of the attempts to reduce fly populations to an economically significant level was never ascertained because follow-up studies did not ensue. Surveys carried out in Tucumán between 1991 and 1993, only recovered small numbers of *P. videmmiae* from *C. capitata* pupae [39]. This pteromalid species was earlier found parasitizing *C. capitata* pupae in the NW province of La Rioja between 1988 and 1990 [46]. Three possible explanations for the presence of *P. vindemmiae *in La Rioja can be drawn: (1) fruit infested with *C. capitata *or *A. fraterculus *larvae parasitized by *P. vindemmiae *could have been moved from Tucumán or Catamarca to La Rioja; (2) *P. vindemmiae *became established in Tucumán spread to La Rioja; (3) *P. vindemmiae *could have been released in La Rioja without official knowledge.

Although *P. vindemmiae* is one of the parasitoid species that has been introduced and recovered in Argentina, it cannot be recognized as part of the group of exotic species introduced to the American continent. This parasitoid is a polyphagous and cosmopolitan species but was also extensively cultured and widely released to combat various tephritid pests [47,48] and synanthropic flies, e.g., in poultry sheds [49]. Originally, *P. vindemmiae* was introduced from West Africa and India to the Hawaiian Islands, and it was then redistributed in Latin America [15]. Its occurrence in 11 American countries [37] can be attributed to three possible causes: (1) a direct result of these purposeful introductions, (2) a synanthropic association, or (3) a simple reflection of its natural distribution. For example, *P. vindemmiae* was previously reported in Tucumán under other scientific names, including *Pachycrepoideus tucumanus* Blanchard [11] and in Mendoza and in Buenos Aires as *Pachycrepoideus dubius* Ashmead [50]. Both scientific names are synonyms of *P. vindemmiae* [51]. Therefore, *D. longicaudata* and *A. indica*, both of which are native species of southeast Asia, are the only confirmed exotic parasitoids currently established in Argentina.

The most recent introduction of parasitoids into Argentina for fruit fly biocontrol occurred in 1999. Two braconid parasitoid species, *Diachasmimorpha tryoni* (Cameron) and *D. longicaudata*, were introduced from México with the purpose of revisiting the use of natural enemies against both *C. capitata* and *A. fraterculus* in the fruit-growing areas of NW and NE Argentina. The original stock consisted of 500 *C . capitata* pupae parasitized by *D. tryoni* and 500 *Anastrepha ludens* (Loew) pupae parasitized by *D. longicaudata *obtained from the Biological Control Laboratory of Mexico’s Moscamed-Moscafrut National Program. All of the pupae were obtained from irradiated *C. capitata* and *A. ludens* larvae [52]. Initially, both parasitoid species were successfully colonized at the CIRPON laboratory on larvae of a wild *C. capitata* strain [53]. In 2005, the parasitoid colony reared on *C. capitata* was transferred to the Biological Control Division of the Pilot Plant of Industrial Microbiological Processes and Biotechnology (PROIMI), and a second colony of *D. longicaudata *was established on *A. fraterculus*. Shipments of *D. longicaudata* specimens from PROIMI’s laboratory to other institutions of technological development in Argentina occurred between 2005 and 2008. The first shipment of parasitoids was sent to INTA in Castelar, Buenos Aires province [54,55]. At this institution, the genetic sexing Cast-191 strain of *C. capitata*, based on a mutation in the *sw *gene that affects the rate of fly development [56], was successfully evaluated as a potential host for rearing *D. longicaudata* [55]. The last significant shipments of *D. longicaudata* specimens were sent to the BioPlanta San Juan facility (central-western Argentina) between April and December 2008 [57].

### 3.2. Current Status

Biological control has recently been incorporated as a tool that is complementary to the fruit fly control and eradication practices currently deployed in the fruit-growing areas of San Juan. The Indo-Pacific species *D. longicaudata* is being mass-reared at the BioPlanta San Juan facility with the objective of mass-releasing this species against *C. capitata*. Certain characteristics of both the biology and ecology of *D. longicaudata* as well as the development of efficient techniques for mass rearing and augmentative releases of this parasitoid in Hawaii [58] and México [59] are considered suitable for selecting it for future augmentative biological control programs in Argentina. Among these traits, the adaptability of *D. longicaudata* to the different environments into which it has been introduced [15,37], its capacity for successful development on the larvae of either *A. fraterculus* or *C. capitata* [37], its host-finding ability at different host-densities on a wide variety of fruit species at canopy and ground levels [60], and its efficacy as a biocontrol agent of several fruit fly species, including *Bactrocera dorsalis* (Hendel) [58], *C. capitata* [61], and *Anastrepha* spp. [13,14,16], are considered the most relevant.

The BioPlanta San Juan is the first facility in Argentina to produce fruit fly parasitoids on a large scale. As a result of this production, a set of laboratory and field-cage experiments and large-scale field trials are being conducted to evaluate (1) the quality of *D. longicaudata* adults reared on larvae of a genetic sexing strain of a temperature-sensitive lethal (*tsl*) Vienna 8 *C. capitata *strain and (2) the effectiveness of the parasitoid once released under semi-arid conditions in ecologically isolated fruit-growing areas of San Juan. At present, approximately 200,000 *D. longicaudata* adults are produced weekly in the BioPlanta San Juan facility using *C. capitata* larvae of the tsl Vienna 8 strain. Due to the high level of parasitoid production, from February 2012 to the present, pilot augmentative releases of parasitoids focused on commercial fig orchards that are heavily infested with *C. capitata* larvae (>50%) in rural areas of San Juan. Parasitoids are released weekly using a ground release system at a density of approximately 5,200 parasitoids per hectare and at a sex ratio of 2:1 (female:male). A substantial reduction in the level of fruit infestation has been achieved to date (F. Murúa, pers. comm.). The, a production of five million *D. longicaudata* wasps per week is planned in a second phase. The goal of ProCEM-San Juan is to use augmentative biological control in combination with sterile fly releases in all fruit production areas of the province to achieve suppression or selected eradication of *C. capitata* populations [57].

In addition to mass rearing of *D. longicaudata* in the BioPlanta San Juan facility, detailed studies on the bioecology of indigenous parasitoid species as well as of the exotic species *D. longicaudata* are ongoing at PROIMI in Tucumán province. The main objective of this research is to assess which parasitoid species is the most suitable for augmentative biological control of both *C. capitata* and *A. fraterculus* in the expanding commercial citrus production areas of NW and NE Argentina, where there are variable climatic and host density conditions. In both citrus-growing regions, ProCEM is planning to carry out integrated fruit fly management programs on an area-wide basis in the coming years incorporating parastioid releases.

In this context, ongoing research in the PROIMI insectary involves assessing the suitability of four indigenous parasitoids species, *Aganaspis pelleranoi *(Brèthes), *Opius bellus* Gahan, *D. crawfordi*, and *Coptera haywardi* Loiácono, for mass rearing on larvae of either *C. capitata *or *A. fraterculus* as part of an augmentative release program targeting both tephritid fruit fly species. To obtain new biological information on these four native parasitoid species, population and reproductive parameters examined via standard life tables as well as parasitism and adult parasitoid emergence rates, sex ratios, and pre-oviposition and oviposition periods are currently being studied under laboratory and field-cage conditions using *A. fraterculus* and *C. capitata* as hosts. In addition, detailed survival analyses through failure time approaches are also being developed [34]. Such information is critical when attempting to select potential candidate species for fruit fly biological control [33,58], because it allows researchers to establish ideal mass-rearing conditions, define efficient release times, and predict survival in the field aimed at determining the proper timing of release intervals to maximize the impact of the parasitoid on the target pest populations [36].

In studies on *D. longicaudata*, the following four issues are currently being examined in the PROIMI insectary: (1) the comparative demography of two *D. longicaudata* strains (reared on larvae of a wild strain of both *C. capitata* and *A. fraterculus*); (2) optimization of laboratory-reared parasitoid yields to achieve and sustain successful mass rearing of these species; (3) the success of *D. longicaudata* females in finding and parasitizing larvae of both *C. capitata* and *A. fraterculus *underfree-foraging fruit choice conditions; and (4) the effect of competitive interactions with indigenous parasitoid species in two particular microhabitats (the tree canopy and the ground under trees).

Simultaneous with the research on indigenous and exotic parasitoids, laboratory and field-cage experiments are being conducted to assess several entomopathogenic fungi strains native to the Yungas forest as potential *C. capitata*/*A. fraterculus* biological control agents.

### 3.3. Future Trends

Five scenarios that broadly impact the development and implementation of fruit fly biological control in Argentina can be delineated. Firstly, the successful establishment of a mass rearing protocol for *D. longicaudata* in the BioPlanta San Juan facility lead to the proposal of this exotic parasitoid as the main candidate for augmentative biological control of *C*. *capitata *in the irrigated fruit-producing valleys in semi-arid areas of San Juan. For this reason, mass production of *D. longicaudata* may lead not only to local use but also to future augmentative area-wide releases of parasitoids in other fruit-growing areas of the Cuyo region, including the provinces of La Rioja and Mendoza. In such semi-arid central-western regions of Argentina, the natural rates of parasitism on *C*. *capitata *are either extremely low or nil [46]. Nevertheless, studies on the bioclimatic requirements of *D. longicaudata* as well as post-release monitoring and assessment of parasitoid efficacy are still needed in these fruit-growing areas.

Secondly, *D. longicaudata* is a potential candidate for use in augmentative releases in citrus crop-growing areas in the subtropical regions of northern Argentina. Efforts to control *A. fraterculus*/*C. capitata* in commercial citrus crops will mainly focus on controlling populations in wild vegetation areas surrounding fruit crops, where feral exotic fruits are highly abundant. From these areas, both pestiferous tephritid species expand to attack commercial orchards [5,25,26]. *Diachasmimorpha longicaudata* is particularly common on large-sized, exotic commercial fruits in Veracruz, Mexico [62,63,64] and Florida, USA [16,65] and can attack both *C. capitata *and *A. fraterculus* larvae [66]. Therefore, mass releases of *D. longicaudata *in abandoned citrus orchards and in vegetation patches containing feral citrus could contribute to the suppression of both *C. capitata* and *A. fraterculus*. Recent field-cage studies have shown that *D. longicaudata* significantly contributed to medfly mortality on infested oranges and grapefruits [67]. These exotic commercial fruits are known to hinder the activity of native braconid parasitoid species recorded in the rain forests of northern Argentina [25]. 

Thirdly, introductions of egg parasitoids that attack *C. capitata* regardless of fruit size seem particularly well suited for almost all of Argentina’s commercial fruit production regions. For instance, Afrotropical parasitoid species such as *F. ceratitivorus* and *F*. *caudatus*, which are natural enemies of *C. capitata* in northern Africa [20], are potential agents for medfly biological control [18,19,68]. Furthermore, Indo-Pacific parasitoid species such as *F. arisanus* can be successfully mass-reared [58,69,70,71] and are capable of suppressing *C. capitata* populations in Hawaii [17] and Guatemala [72]. The egg-parasitoid *F. arisanus* could be reintroduced into Argentina and mass reared in the BioPlanta San Juan facility with augmentative releases of *F. arisanus* focusing on areas where *C. capitata* hosts are abundant.

Fourthly, releases of indigenous parasitoids in vegetation areas adjacent to commercial fruit groves during periods of high host availability [23] could enhance natural populations of parasitoids. This strategy could contribute greatly to the establishment of low *A. fraterculus* prevalence areas in northern citrus-growing regions. The selection of indigenous parasitoid species for releases should be carried out considering native parasitoid guilds, target pest fly species, host fruit species, the foraging behavior of each parasitoid species, and the feasibility of rearing. Of the four native parasitoid species that were successfully colonized in the PROIMI insectary, *A. pelleranoi*, *O. bellus* and *C. haywardi* represent good candidates. Of these three parasitoid species, the figitid *A. pelleranoi* and the diapriid *C. haywardi* have very interesting attributes. These two species are among the few neotropical parasitoid species associated with *A. fraterculus* that are able to attack and successfully develop on *C. capitata*. In addition, *A. pelleranoi* preferentially forages on the ground and can therefore attack tephritid larvae on fallen fruits [25,73]. Furthermore, *C. haywardi* only attacks pupae of tephritid flies [74], and it has recently been cited as a viable biocontrol agent for augmentative releases targeting *Anastrepha* spp. [33,36,75,76].

Finally, in rural areas of the Argentinan subtropical northern region in which indigenous parasitoids of *A*. *fraterculus* are well established in all native fruit species and in small feral exotic fruits [25,27], management strategies could be used to conserve natural populations of the parasitoids. Instead of removing native host plants, which represent natural parasitoid reservoirs, they should be preserved. For this purpose, it would be wise to at least consider the possibility of maintaining patches with interspersed native vegetation within areas where there is large-scale agricultural production, as suggested by Aluja *et al.* [77] for tropical regions of México. Both Argentinean subtropical rain forests, belonging to the neotropical region, undoubtedly represent a valuable source of existing natural enemies and new potential biocontrol agents, such as entomopatogenic fungi, that could be employed to abate populations of *A. fraterculus* as well as *C. capitata*.

## 4. Conclusions

Biological control is a viable strategy for the suppression and management of both *A. fraterculus* and *C. capitata* in many of the fruit-growing areas of Argentina. The use of biological control under the expanded ProCEM will supply additional tools to ensure environmental protection and commercial sustainability through reduced application of conventional insecticides for the control of both pestiferous fruit fly species. However, two significant issues should be considered before the implementation of a long-term biological control program. 

Firstly, the control of both tephritid species with parasitoids alone is not likely to provide the level of economic control required. Even though parasitism levels as high as 90% are observed, fruit infestation rates of 5% are usually considered economically unacceptable in fruit cropping systems [15,23]. Furthermore, the expanded ProCEM has the objective of reducing fruit damage levels from 13 to 0.5% and maintaining them at this level over a long period in the citrus-growing areas of northern Argentina [2]. Achieving this goal would reduce the risk of reinfestation by medflies in protected fruit-producing regions with a fruit fly-free or low prevalence status through trade in infested citrus fruits in these regions. Thus, biorational management strategies on an area-wide basis in combination with the conservation of indigenous parasitoids or augmentation of both exotic and native parasitoids are needed to suppress *A. fraterculus*/*C. capitata* populations.

Secondly, biological control strategies that are appropriate for the irrigated fruit-producing areas of the Cuyo region do not necessarily apply to the citrus-growing areas in northern Argentina. The fruit fly control situation in the latter area is complicated by several major interrelated factors, including (1) the presence of two pest species in orchards and also in surrounding non-commercial orchards and both unperturbed and disturbed wild vegetation areas; (2) a favorable warm humid climate; (3) the production of numerous pest generations per year; and (4) fruit fly populations that build up in feral fruit hosts mainly located in patches with perturbed native vegetation near commercial citrus crops. Therefore, biological control actions must be adapted to each Argentinean fruit-producing region according to these factors. A key aspect in achieving a well-planned biological control program is the integration of actions between ProCEM, local governments, small and large growers, and national research centers. A particularly good example of this type of integration is the pilot augmentative biological control program recently implemented against medfly in fruit-growing semi-arid areas of San Juan [57].
